# Supplementary material for: Structural Characterization and Heparanase Inhibitory Activity of Fucosylated Glycosaminoglycan from Holothuria floridana
Source: Mar Drugs. 2021 Mar 18;19(3):162. doi: 10.3390/md19030162 (PMC8003118; doi:10.3390/md19030162)
Supplement: Supplementary file 1 [file marinedrugs-19-00162-s001.pdf]

marine drugs

MDPI

Supporting Information

Structural Characterization and Heparanase Inhibitory Activity of Fucosylated Glycosaminoglycan from *Holothuria floridana*

Xiang Shi[1#], Ruowei Guan[1#], Lutan Zhou[2], Zhichuang Zuo[1], Xuelin Tao[1], Pin Wang[1], Yanrong Zhou[1], Ronghua Yin[1], Longyan Zhao[1,3*], Na Gao[1,4*], Jinhua Zhao[1]

1 School of Pharmaceutical Sciences, South-Central University for Nationalities, Wuhan 430074, China; sx20200807@163.com (X. S.); ruoweiguan13@163.com (R. G.); zuo19951224@163.com (Z. Z.); txl6361@163.com (X.T.); wangpin1994@163.com (P. W.); zyr17577300547@163.com (Y. Z.); yinronghua@mail.kib.ac.cn (R. Y.); gn2008.happy@163.com (N. G.); zhaojinhua@mail.kib.ac.cn (J. Z.)

2 State Key Laboratory of Phytochemistry and Plant Resources in West China, Kunming Institute of Botany, Chinese Academy of Sciences, Kunming 650201, China; zhoulutan@mail.kib.ac.cn (L. Z.)

3 Institute of Marine Drugs, Guangxi University of Chinese Medicine, Nanning 530200; China; longyanzhao@gmail.com (L. Z.)

4 National Demonstration Center for Experimental Ethnopharmacology Education, South-Central University for Nationalities, Wuhan 430074, China; gn2008.happy@163.com (N. G.)

# These authors contributed equally to this work.

* Correspondence: longyanzhao@gmail.com (Longyan Zhao); gn2008.happy@163.com (Na Gao)

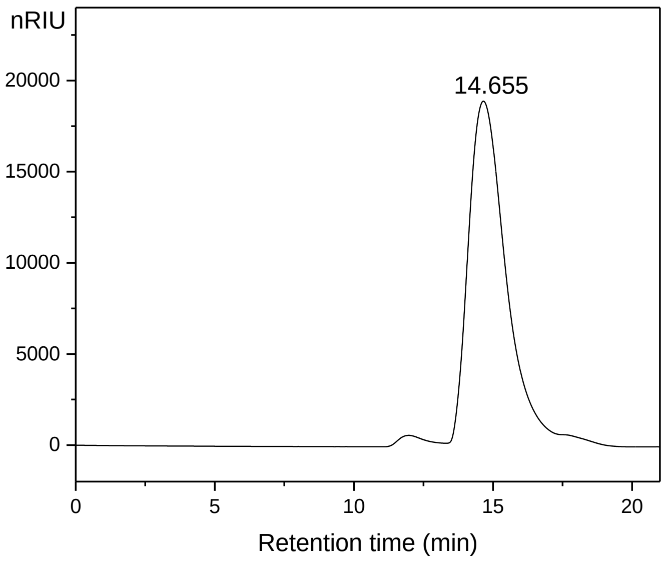

Figure S1. HPLC of 2.0 M fraction (HfFG-2M)

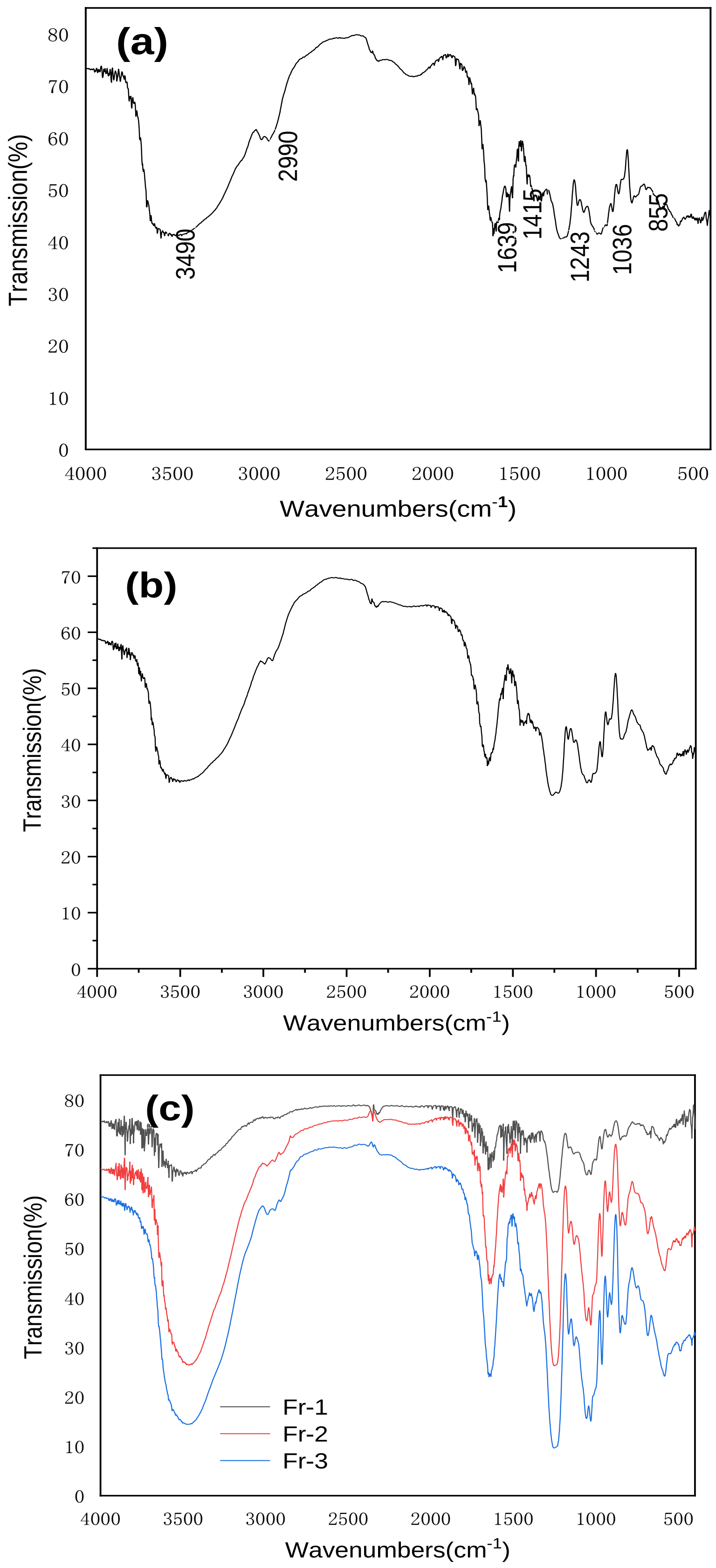

Figure S2. IR spectra of HfFG (**a**), dHfFG (**b**), Fr-1, Fr-2 and Fr-3 (**c**)

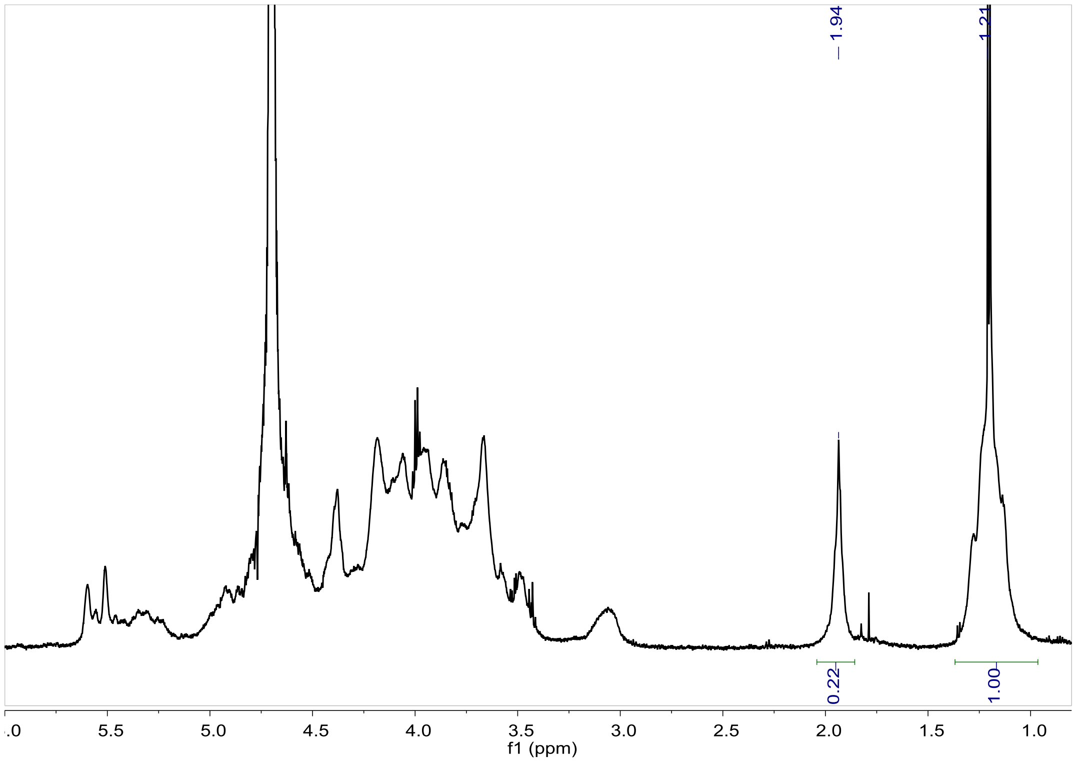

Figure S3. ¹H NMR spectrum of partially deacetylated HfFG

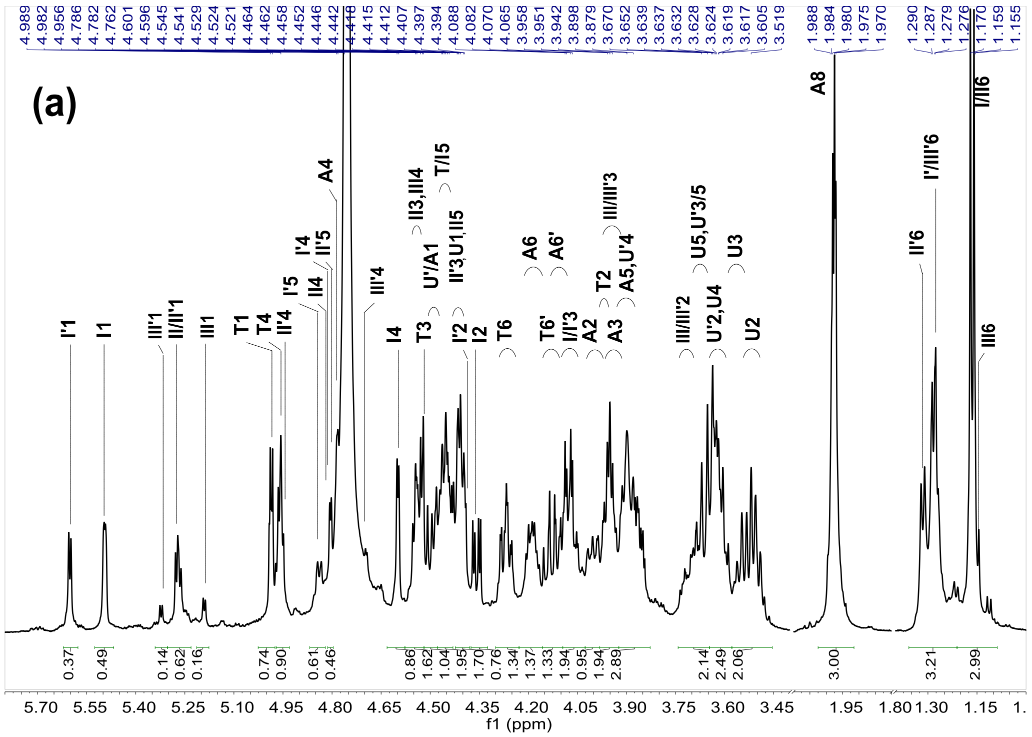

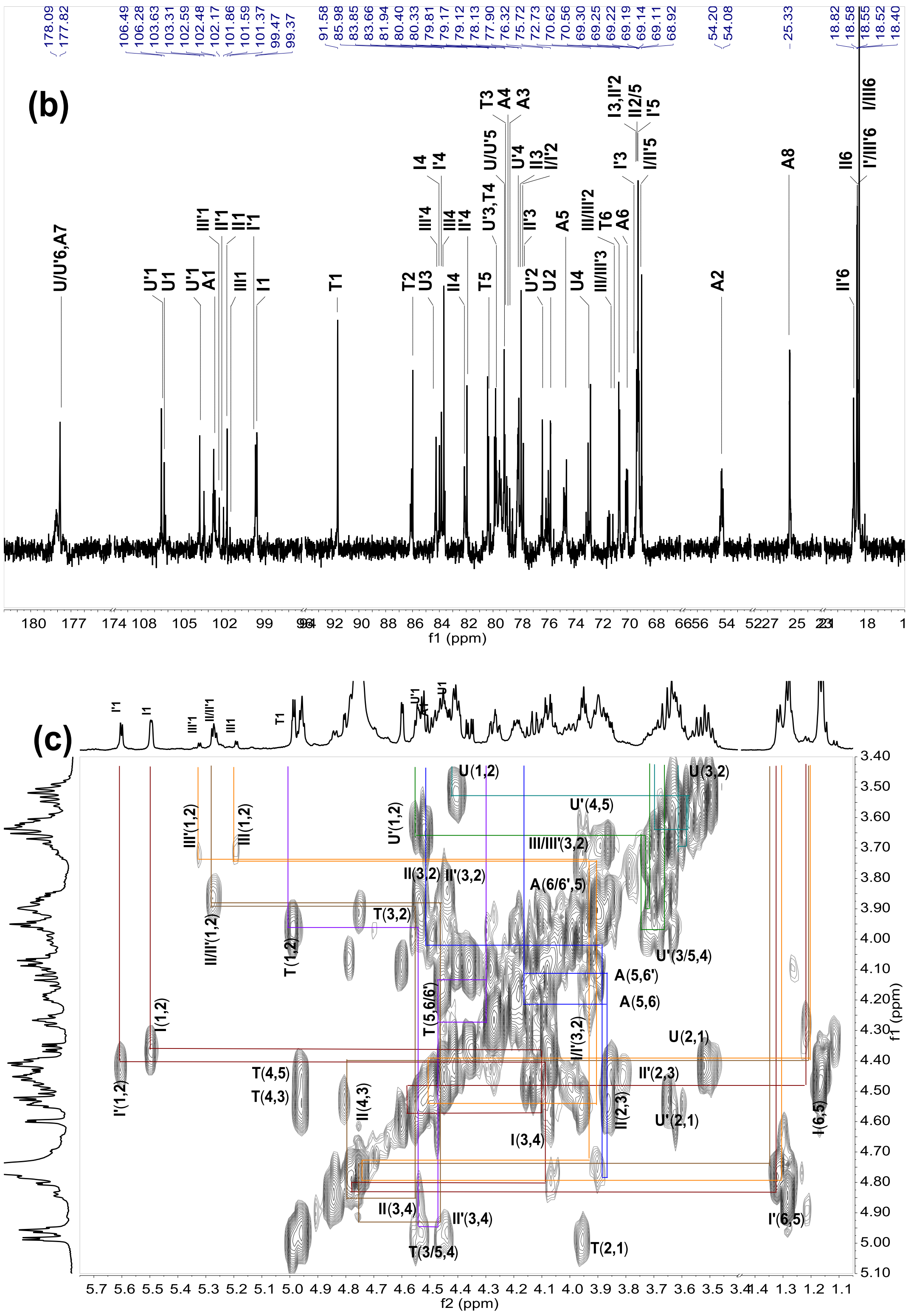

(b)
178.09
177.82
106.49
106.28
103.63
103.31
102.59
102.48
102.17
101.86
101.59
101.37
99.47
99.37
91.58
85.98
83.85
83.66
81.94
80.40
80.33
79.81
79.17
79.12
78.13
77.90
76.32
75.72
72.73
70.62
70.56
69.30
69.25
69.22
69.19
69.14
69.11
68.92
54.20
54.08
25.33
18.82
18.58
18.55
18.52
18.40
U/U'6,A7
U'1
U1
U'1
A1
III'1
II'1
II1
I'1
III1
I1
T1
T2
U3
III'4
I4
I'4
II4
II'4
II4
T3
A4
A3
U/U'5
U'3,T4
T5
U'4
II3
I/I'2
II'3
U'2
U2
A5
U4
III/III'2
III/III'3
T6
A6
I3,II'2
II2/5
I'5
I'3
I/II'5
A2
A8
II6
I/III6
I'/III'6
II'6
f1 (ppm)
(c)
I'1
I1
III'1
II/II'1
III1
T1
U'1
U1
U(1,2)
U(3,2)
U'(4,5)
U'(1,2)
III'(1,2)
III(1,2)
III/III'(3,2)
II(3,2)
II'(3,2)
A(6/6',5)
II/II'(1,2)
T(3,2)
T(1,2)
U'(3/5,4)
A(5,6')
A(5,6)
I(1,2)
T(5,6/6')
U(2,1)
I/I'(3,2)
II'(2,3)
T(4,5)
T(4,3)
I'(1,2)
II(4,3)
II(2,3)
U'(2,1)
I(6,5)
I(3,4)
II(3,4)
II'(3,4)
I'(6,5)
T(3/5,4)
T(2,1)
f2 (ppm)
f1 (ppm)

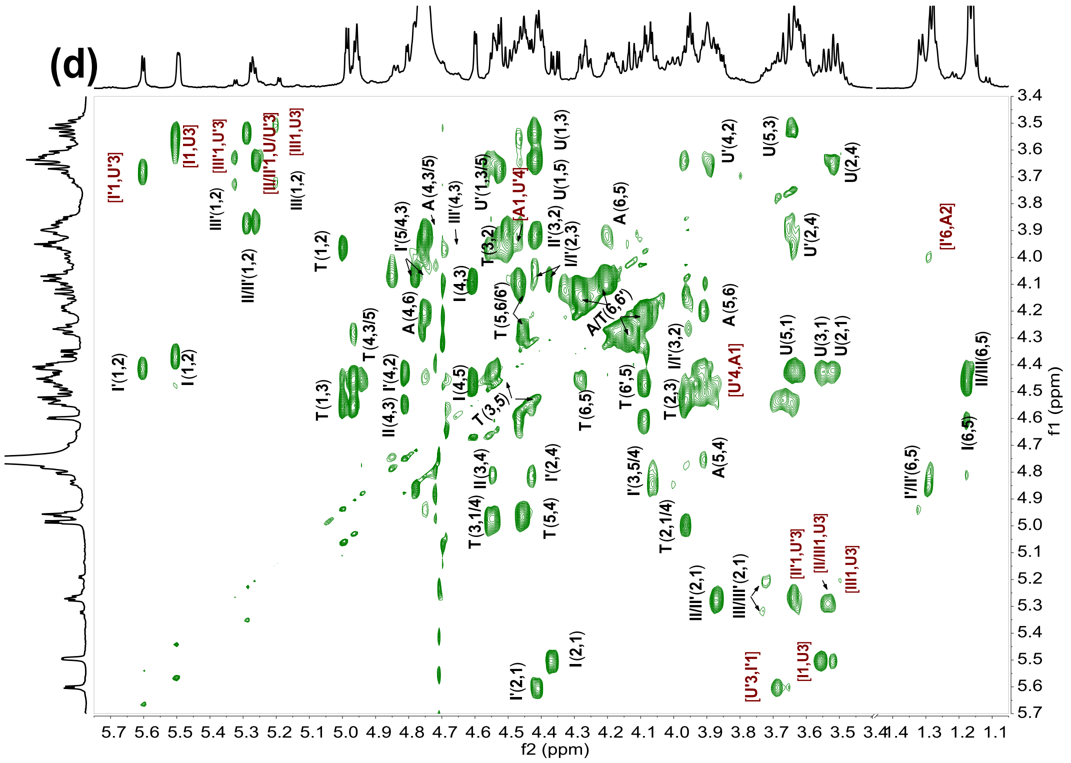
(d)
f2 (ppm)
f1 (ppm)

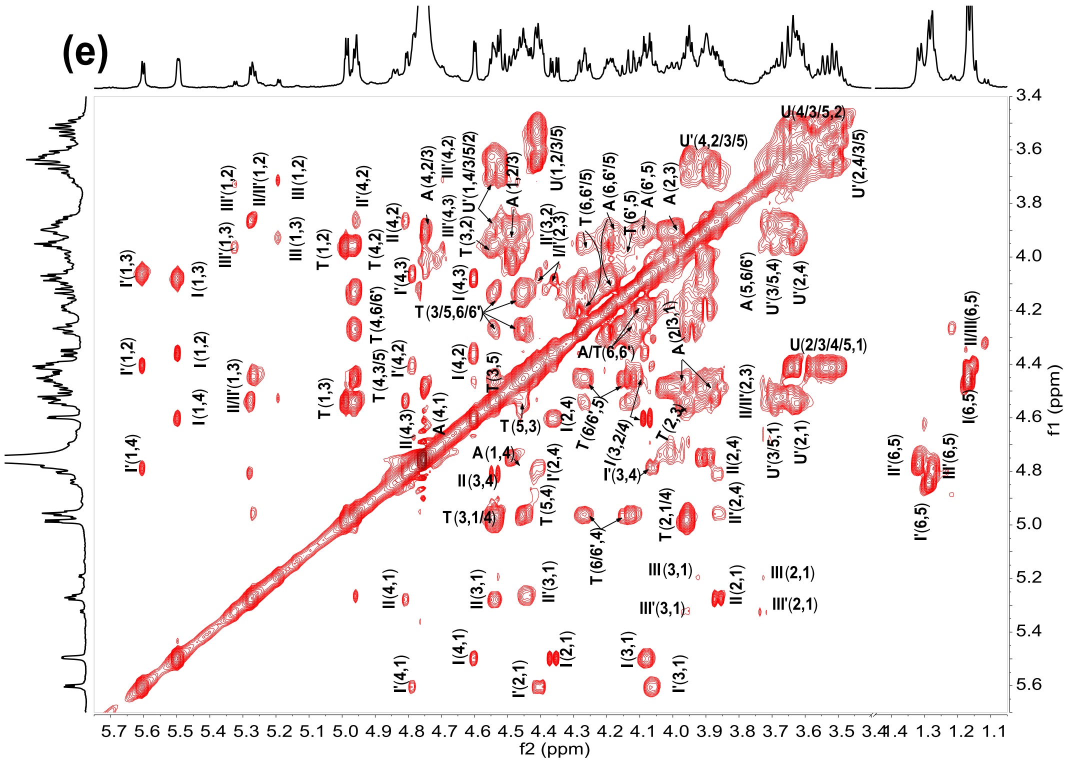
(e)
f2 (ppm)
f1 (ppm)

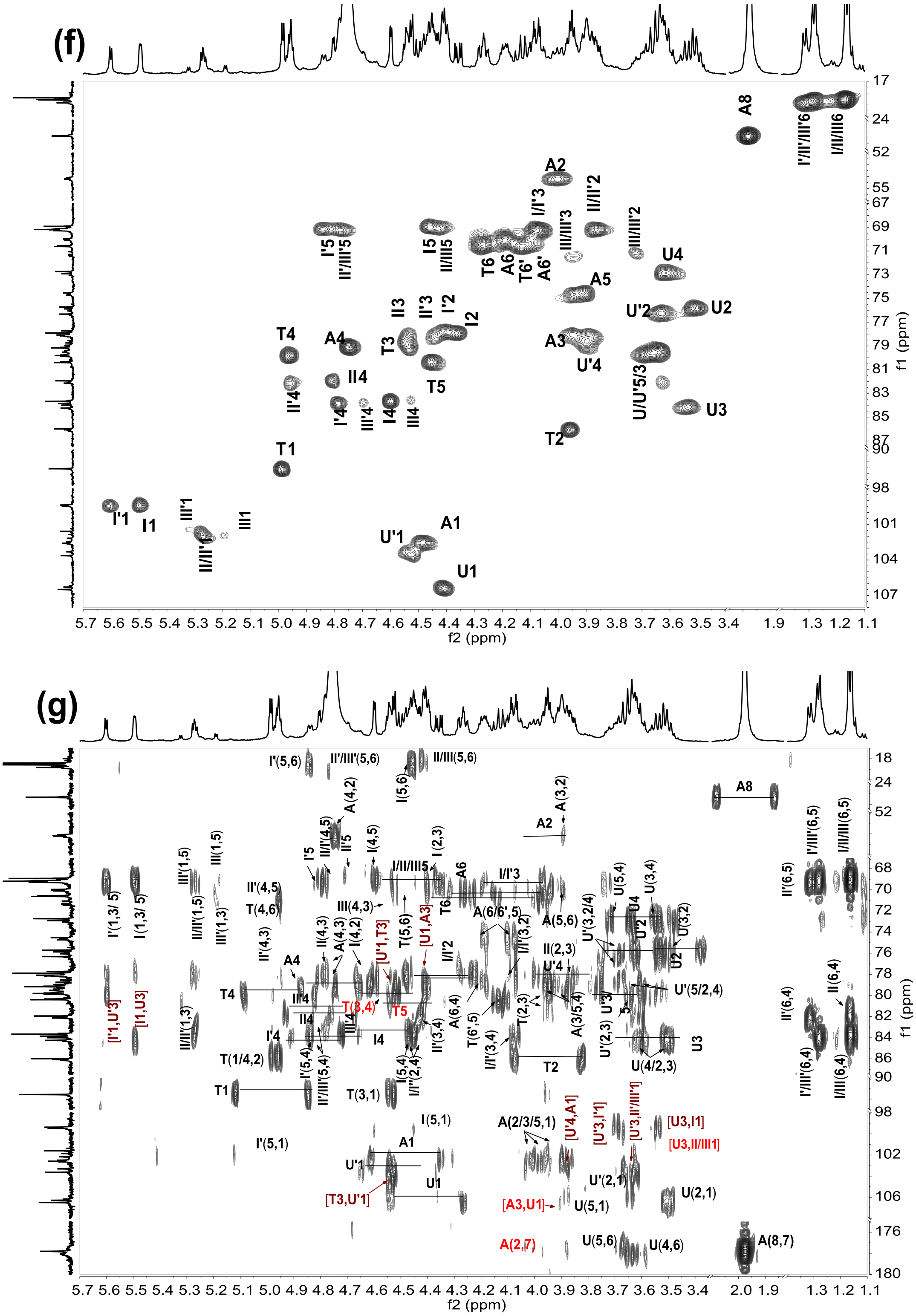

Figure S4. ^{1}H (**a**), ^{13}C (**b**), ^{1}H-^{1}H COSY (**c**), TOCSY (**d**), ROESY (**e**), ^{1}H-^{13}C HSQC (**f**) and HMBC (**g**) spectra of Fr-1

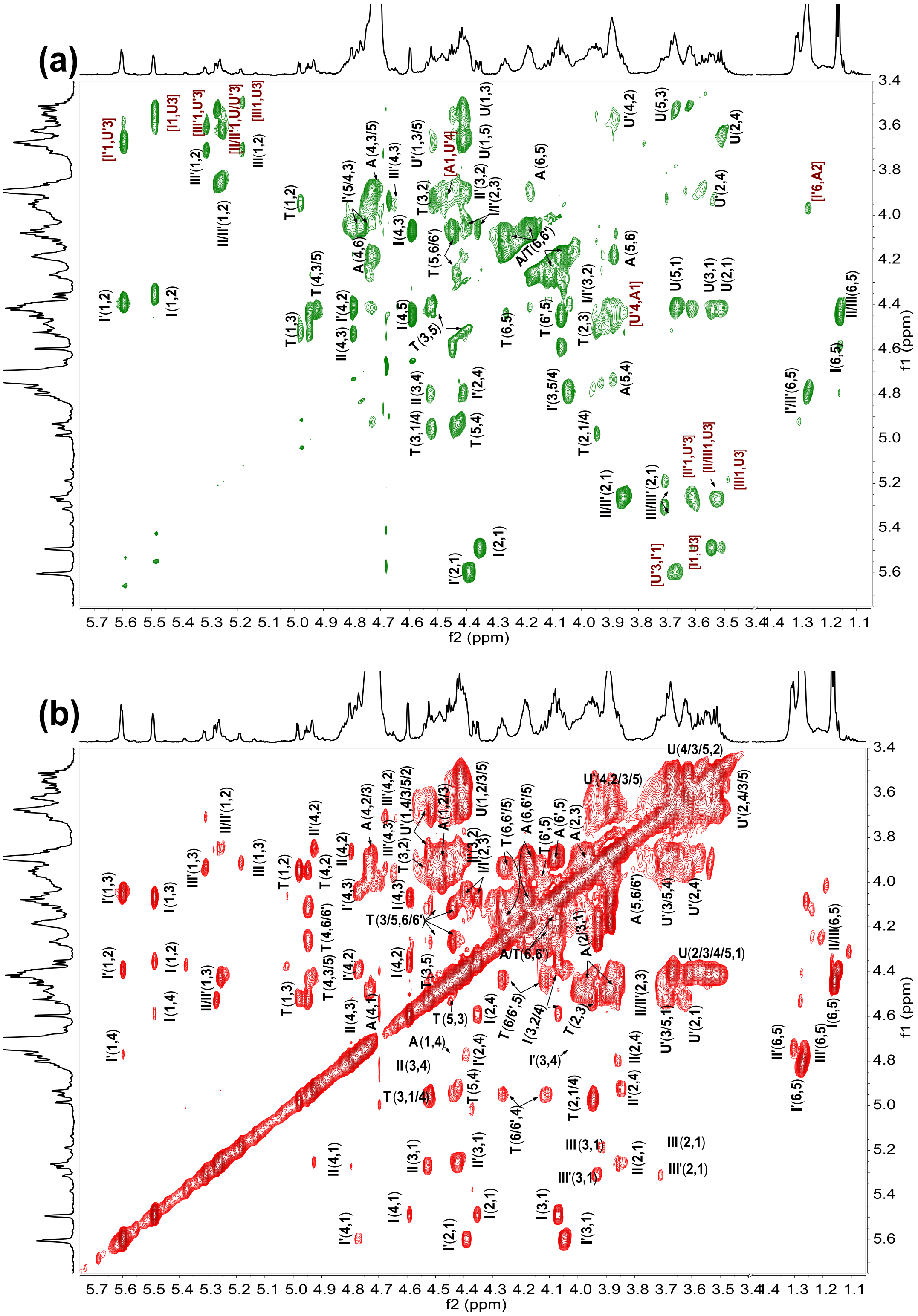

Figure S5. ^{1}H-^{1}H ROESY (**a**) and TOCSY (**b**) spectra of Fr-2

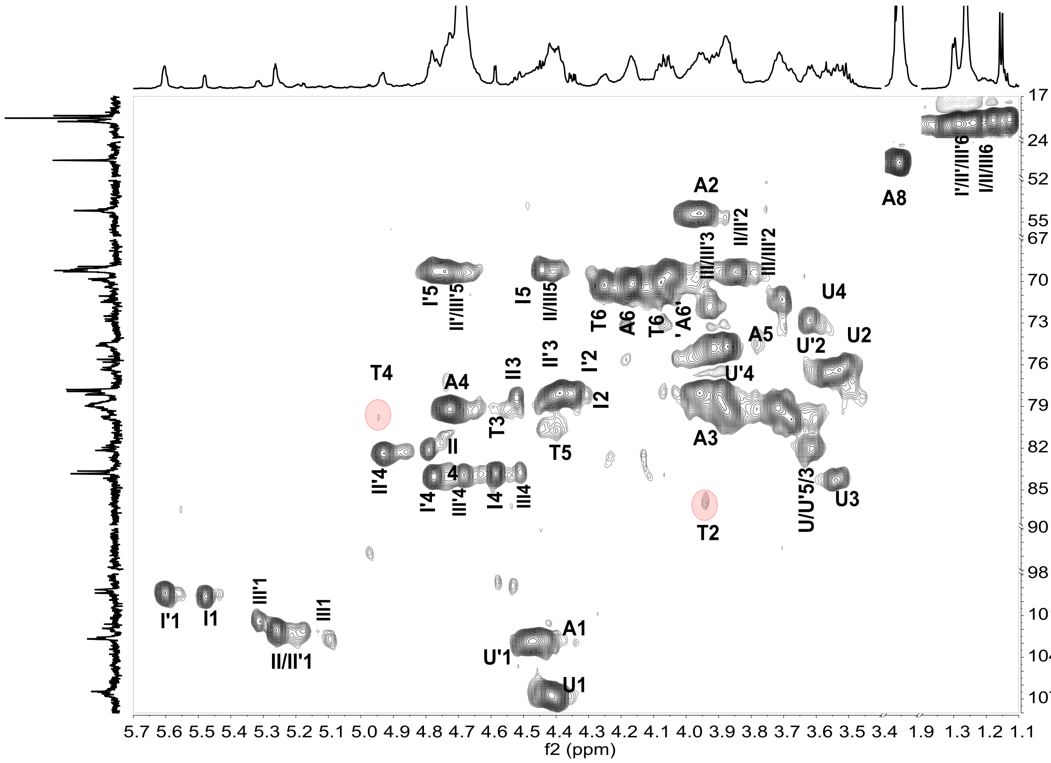

Figure S6. ^{1}H-^{13}C HSQC spectrum of Fr-3

Table S1. $^{1}H/^{13}C$ NMR chemical shift assignments of Fr-1 (800 MHz, D_2O)

Residue		Chemical shift (ppm)								
		H/C	1	2	3	4	5	6	7	8
T	→3)-β-D-anTal$_{4S6S}$-diol	H	4.985	3.954	4.526	4.96	4.455	4.267/4.130		
		C	91.59	85.98	79.14	79.7	80.4	70.63		
U′	→4)-β-D-GlcA-(1→	H	4.533	3.631	3.67	3.9	3.673			
		C	103.64	76.33	79.85	78.15	79.32	177.34		
A	→3)-β-D-GalNAc$_{4S6S}$-(1→	H	4.487	4.003	3.95	4.75	3.906	4.105/4.190		
		C	102.6	54.14	78.9	79.05	74.53	69.97	177.82	25.35
F′ (I′)	α-L-Fuc$_{2S4S}$-(1→	H	5.605	4.404	4.073	4.788	4.842	1.276		
		C	99.48	77.91	69.3	83.67	69.06	18.82		
F′ (II′)	α-L-Fuc$_{3S4S}$-(1→	H	5.267	3.864	4.44	4.951	4.782	1.317		
		C	101.86	69.2	77.74	81.95	68.93	18.76		
F′(III′)	α-L-Fuc$_{4S}$-(1→	H	5.317	3.72	3.943	4.698	4.753	1.269		
		C	101.92	71.29	71.47	84	68.98	18.53		
U	D-GlcA-(1→	H	4.414	3.511	3.544	3.612	3.671			
		C	106.49	75.72	84.25	72.73	79.32	177.82		
F (I)	α-L-Fuc$_{2S4S}$-(1→	H	5.497	4.36	4.083	4.602	4.458	1.167		
		C	99.37	77.91	69.2	83.85	68.93	18.4		
F (II)	α-L-Fuc$_{3S4S}$-(1→	H	5.271	3.86	4.537	4.807	4.407	1.167		
		C	101.6	69.12	78.09	82.14	69.12	18.55		
F (III)	α-L-Fuc$_{4S}$-(1→	H	5.191	3.716	3.943	4.529	4.407	1.143		
		C	101.36	71.29	71.47	83.59	68.98	18.4		
